# Effective Extracellular Volume Fraction Determined by Equilibrium Contrast-Enhanced CT for Differentiating Autoimmune Pancreatitis from Pancreatic Ductal Adenocarcinoma

**DOI:** 10.3390/diagnostics15151845

**Published:** 2025-07-22

**Authors:** Akihiko Kanki, Yoshihiko Fukukura, Hidemitsu Sotozono, Kiyoka Maeba, Atsushi Higaki, Yuki Sato, Akira Yamamoto, Ryo Moriwake, Tsutomu Tamada

**Affiliations:** Department of Radiology, Kawasaki Medical School, 577 Matsushima, Kurashiki City 701-0192, Okayama, Japan; yoshihiko2014jp@gmail.com (Y.F.); kiyo@med.kawasaki-m.ac.jp (K.M.); ahah@med.kawasaki-m.ac.jp (A.H.); yuki310310@gmail.com (Y.S.); jiro@med.kawasaki-m.ac.jp (A.Y.); moriwake@med.kawasaki-m.ac.jp (R.M.); ttamada@med.kawasaki-m.ac.jp (T.T.)

**Keywords:** pancreas, autoimmune pancreatitis, extracellular space, carcinoma, pancreatic ductal

## Abstract

**Background/Objectives:** The aim of this study was to determine whether extracellular volume (ECV) fraction as determined by contrast-enhanced computed tomography (CECT) can help distinguish between autoimmune pancreatitis (AIP) and pancreatic ductal adenocarcinoma (PDAC). **Methods:** Participants comprised 101 patients, including 20 diagnosed with AIP (AIP group), 42 with histologically confirmed PDAC (PDAC group), and 39 without pancreatic disease (healthy group). Contrast enhancement (CE) was calculated as CT attenuation in Hounsfield units [HU] on equilibrium-phase CECT–CT attenuation on pre-contrast CT. The ECV fraction was calculated by measuring the region of interest within the pancreatic region and aorta on pre-contrast and equilibrium-phase CECT. CT measurements were compared among groups. CE and ECV fractions were also compared for diffuse (*n* = 12) and focal or segmental types (*n* = 8). Focal- or segmental-type AIP was defined as the involvement of one or two pancreas segments. Diagnostic efficacy was evaluated through receiver operating characteristic (ROC) analyses. **Results:** CE and ECV fractions differed significantly between the groups (*p* < 0.001 each). CE was significantly higher in the AIP group (56.8 ± 7.9 HU) than in the PDAC group (42.3 ± 17.0 HU, *p* < 0.001) or healthy group (32.2 ± 6.1 HU, *p* < 0.001). ECV fraction was significantly higher in the AIP group (47.2 ± 7.3%) than in the PDAC group (31.7 ± 12.0%, *p* < 0.001) or healthy group (27.5 ± 5.4%, *p* < 0.001). In the AIP group, no significant differences in CE (56.7 ± 8.2 HU vs. 56.9 ± 8.1 HU; *p* = 1.000) or ECV fraction (48.0 ± 5.6% vs. 46.6 ± 8.4%; *p* = 0.970) were seen between diffuse and focal or segmental types. Areas under the ROC curve for differentiating AIP from PDAC were 0.78 for CE and 0.86 for ECV fraction, showing no significant difference (*p* = 0.083). **Conclusions:** ECV fraction might be clinically useful in differentiating AIP from PDAC.

## 1. Introduction

Autoimmune pancreatitis (AIP) was first described in 1995 by Yoshida et al. [1] and represents a subtype of chronic pancreatitis [2,3]. AIP is divided into two types: lymphoplasmacytic sclerosing pancreatitis (type 1 AIP) and idiopathic duct-centric chronic pancreatitis (type 2 AIP). Both types are known to exhibit diffuse and focal types. Their clinical and imaging features overlap with those of pancreatic ductal adenocarcinoma (PDAC) [2,4,5,6]. A previous report suggested that 1.6% of AIP patients underwent pancreaticoduodenectomy on preoperative suspicion of PDAC [7]. Distinguishing between these two entities is clinically crucial due to the completely different pathological conditions involved, resulting in differences in both treatment methods and prognoses [3]. Several reports have attempted to differentiate AIP from PDAC using morphological features and differences in contrast enhancement (CE) on contrast-enhanced computed tomography (CECT) [8,9,10,11], but the accuracy of this method remains poor even for the most experienced radiologists [12]. In addition, CE on CECT lacks quantitative properties because of the dependence on CT scan protocols, patient characteristics, and the vascular distribution in the target tissue [13]. Better imaging techniques are therefore needed to differentiate between these pathologies.

Perfusion CT and dynamic contrast-enhanced (DCE) magnetic resonance imaging (MRI) allow for quantitative analysis of various perfusion or permeability parameters that can reflect tissue characteristics and have been used to differentiate between AIP and PDAC [14,15,16,17]. However, perfusion CT and DCE-MRI require technically complex post-processing after the images are obtained, which may impede routine clinical use. The extracellular volume (ECV) fraction is the total of the extravascular extracellular volume fraction and the intravascular cavity fraction. ECV fraction is easily calculated from the CT attenuation values of pancreatic lesions and the aorta in the pre-contrast and equilibrium phases and haematocrit, and it does not require a long time or specialised hardware or software for additional investigations. ECV fraction as determined from equilibrium CECT or MRI has been used for the assessment of cardiac, hepatic, and pancreatic fibrosis [18,19,20,21,22,23]. ECV fraction has also been reported to a prognostic factor as an alternative to perfusion-CT or DCE-MRI for predicting treatment response to chemotherapy for PDAC and postoperative pancreatic fistula [19,24,25]. Furthermore, ECV fraction has been reported to exhibit a strong correlation with the volume transfer constant (Ktrans) and the extravascular extracellular volume (Ve) as measured by DCE-MRI [26]. However, no reports to date have clarified the use of ECV fraction to differentiate AIP from PDAC. The purpose of this study was to determine whether ECV from CECT can help distinguish AIP from PDAC.

## 2. Materials and Methods

### 2.1. Patients

Institutional review board approval was obtained for this study and the need for informed patient consent was waived based on the retrospective design (approval number: 5633-01).

**Figure 1:**
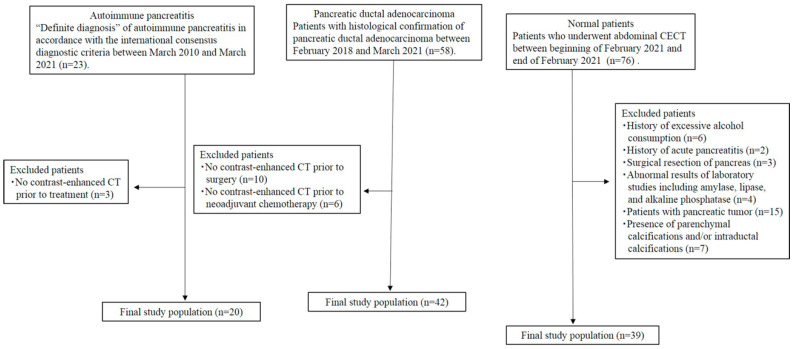
Flow diagram of the study population.

Using our medical records and CT database for the period from the beginning of March 2010 to the end of March 2021, we retrospectively identified 20 consecutive patients (14 men, 6 women; mean age, 64.5 years; range, 45–74 years) with AIP (AIP group) who met the following criteria: patients who were categorised as having a “definite diagnosis” of AIP based on a combination of histological and imaging data and therapeutic response to corticosteroids in accordance with the international consensus diagnostic criteria [27] and patients who underwent abdominal CECT prior to treatment for AIP (Figure 1).

A total of 42 consecutive patients (28 men, 14 women; mean age, 70.1 years; range, 47–88 years) with PDAC (PDAC group) were finally included in this study between February 2018 and March 2021 based on the following inclusion criteria: patients who were pathologically diagnosed by fine-needle aspiration biopsy (*n* = 9) or resection (*n* = 33) and patients who underwent abdominal CECT prior to treatment for PDAC. To select a healthy pancreas group, we also searched the CT database using the search terms “pancreas AND abdominal CECT” and identified 76 patients between the beginning of February 2021 and the end of February. The study coordinator retrospectively reviewed their medical records and abdominal CECT. Patients were excluded for the following reasons: a history of excessive alcohol consumption (at least 60 g/day), acute pancreatitis, or surgical resection of the pancreas; abnormal results from laboratory studies, including amylase, lipase, and alkaline phosphatase; or the presence of pancreatic tumour, parenchymal calcifications, or intraductal calcifications, or abnormalities of the pancreatic duct (pancreatic duct dilatation and strictures). A total of 39 consecutive patients (22 men, 17 women; mean age, 63.1 years; range 25–94 years) were finally included in the study as healthy patients. Patient characteristics are presented in Table 1.

### 2.2. CT Protocol

CT images were obtained using one of four multidetector-row CT devices (Aquilion Prime SP, Aquilion Prime SP/i Edition, Aquilion 64; Canon Medical Systems, Tokyo, Japan, or Lightspeed Ultra 16; General Electric Medical Systems, Milwaukee, WI, USA). All patients received iodinated non-ionic contrast agent at a dose of 600 mg I/kg patient body weight with a fixed injection duration of 30 s using an automated power injector (Nemoto Kyorindo, Tokyo, Japan). Contrast agent was injected through a 20-gauge plastic intravenous catheter placed in an antecubital vein. After obtaining pre-contrast CT images, CECT images were acquired with time delays of 40 s, 70 s, and 210 s during the pancreatic, portal venous, and equilibrium phases, respectively, according to our optimised clinical protocol. Imaging parameters for all scanners are shown in Table 2.

### 2.3. Image Analysis

**Figure 2:**
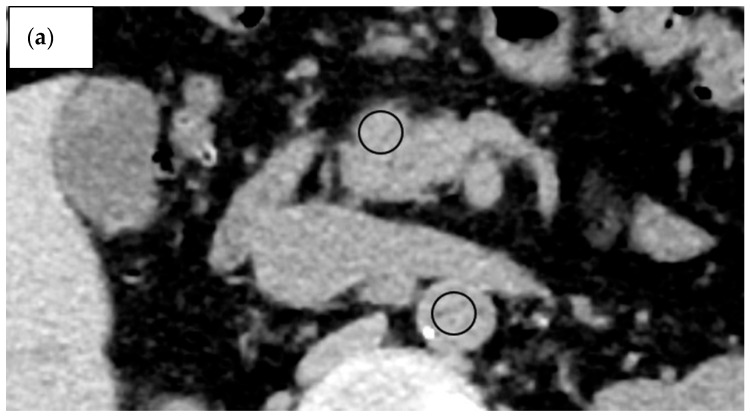

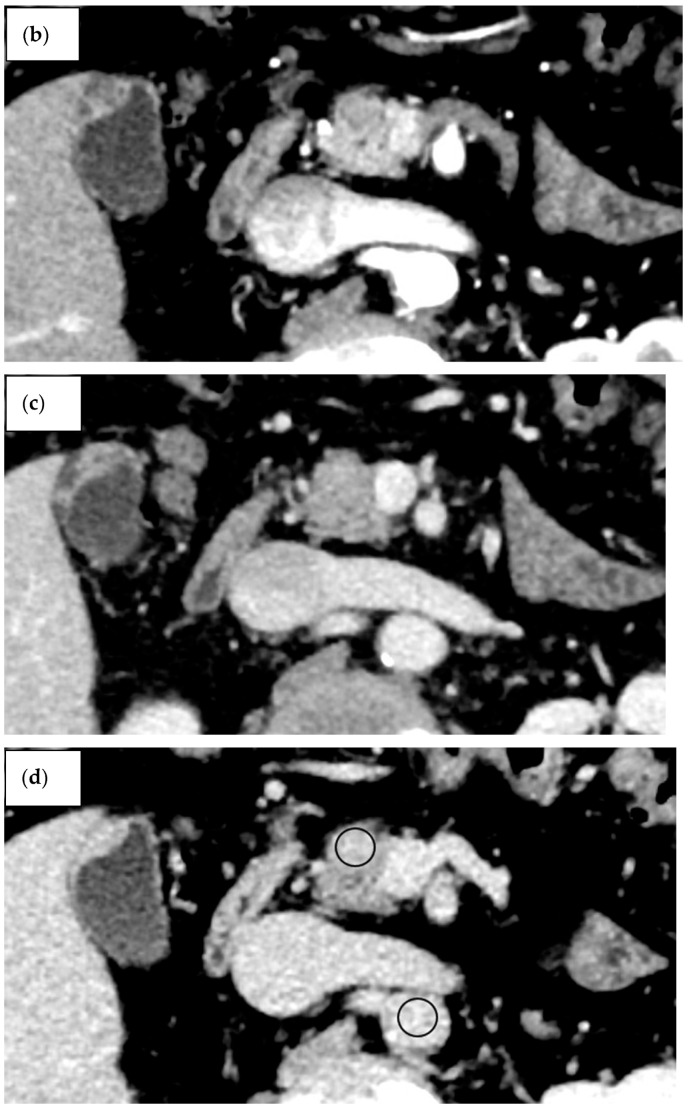

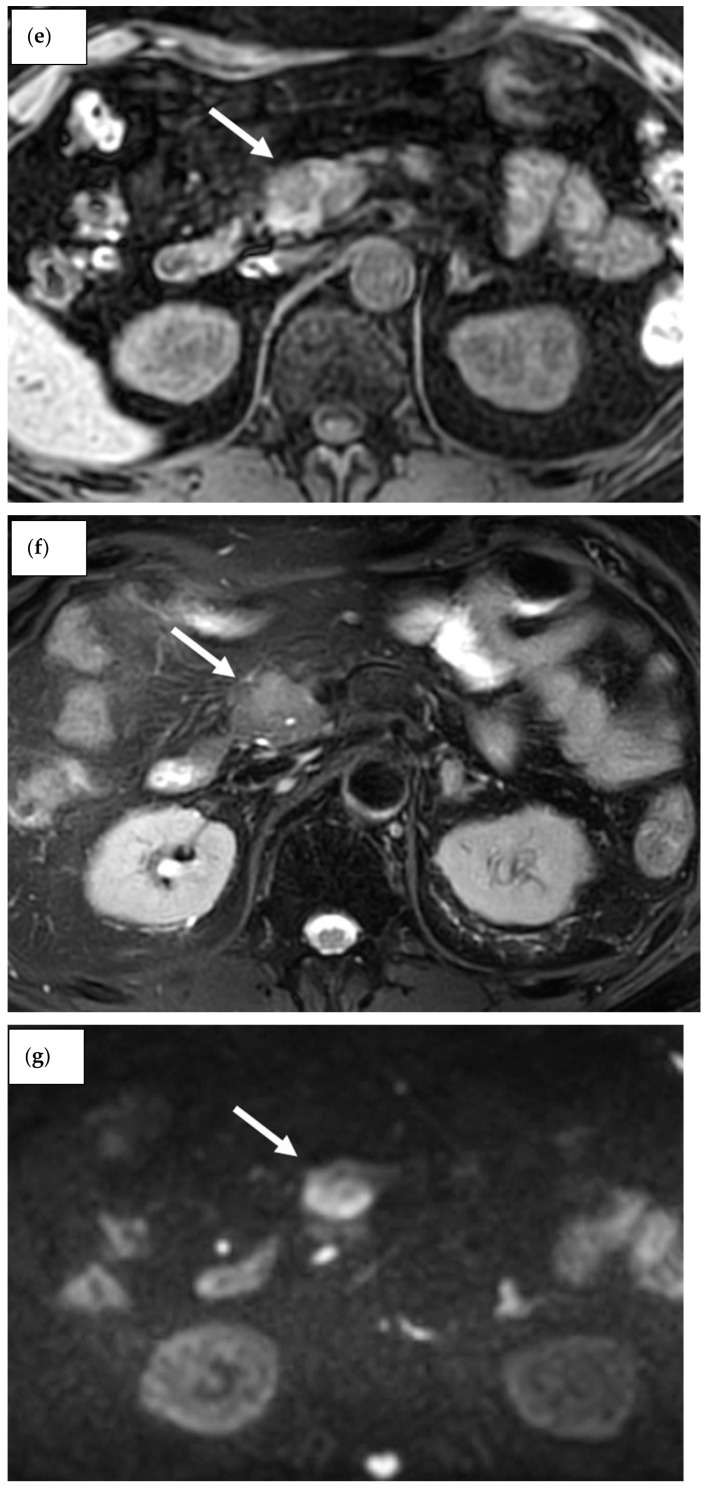

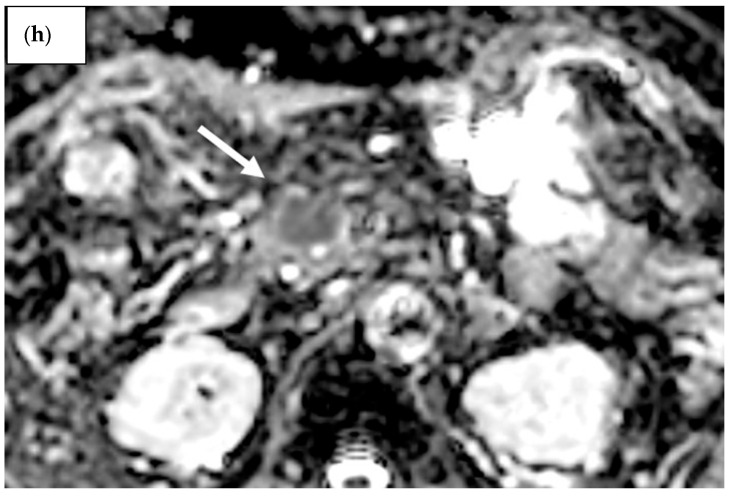
A 59-year-old man with autoimmune pancreatitis (AIP). (**a**–**d**) CT images pre-contrast (**a**) and during the pancreatic phase (**b**), portal venous phase (**c**), and equilibrium phase (**d**). Regions of interest (in black) are manually drawn on the AIP region and abdominal aorta on pre-contrast (**a**) and equilibrium-phase (**d**) images. The calculated contrast enhancement was 56.1 HU and the extracellular volume fraction was 55.5%. (**e**) On pre-contrast, the AIP region located in the head of the pancreas shows low signal intensity compared with normal pancreatic parenchyma (arrow). (**f**) T2-weighted image with fat suppression. The AIP region located in the head of the pancreas shows slight signal hyperintensity compared with normal pancreatic parenchyma (arrow). (**g**) Diffusion-weighted imaging (b = 800 s/mm^2^) shows the AIP region with high signal intensity (arrow). (**h**) The apparent diffusion coefficient is 0.89 × 10^−3^ mm^2^/s (arrow).

**Figure 3:**
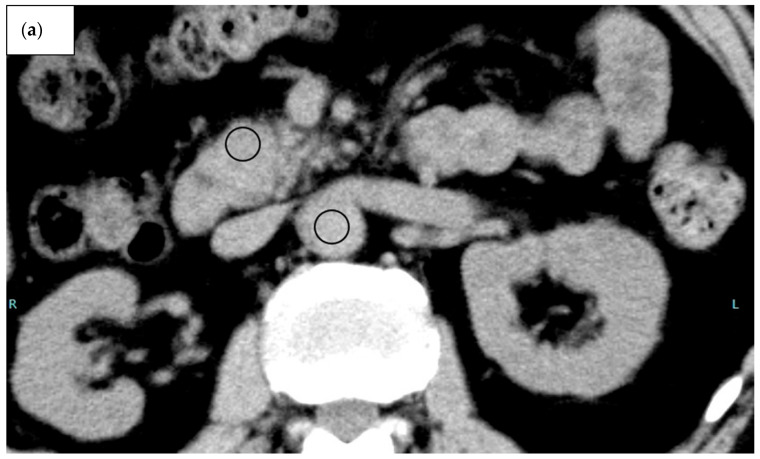

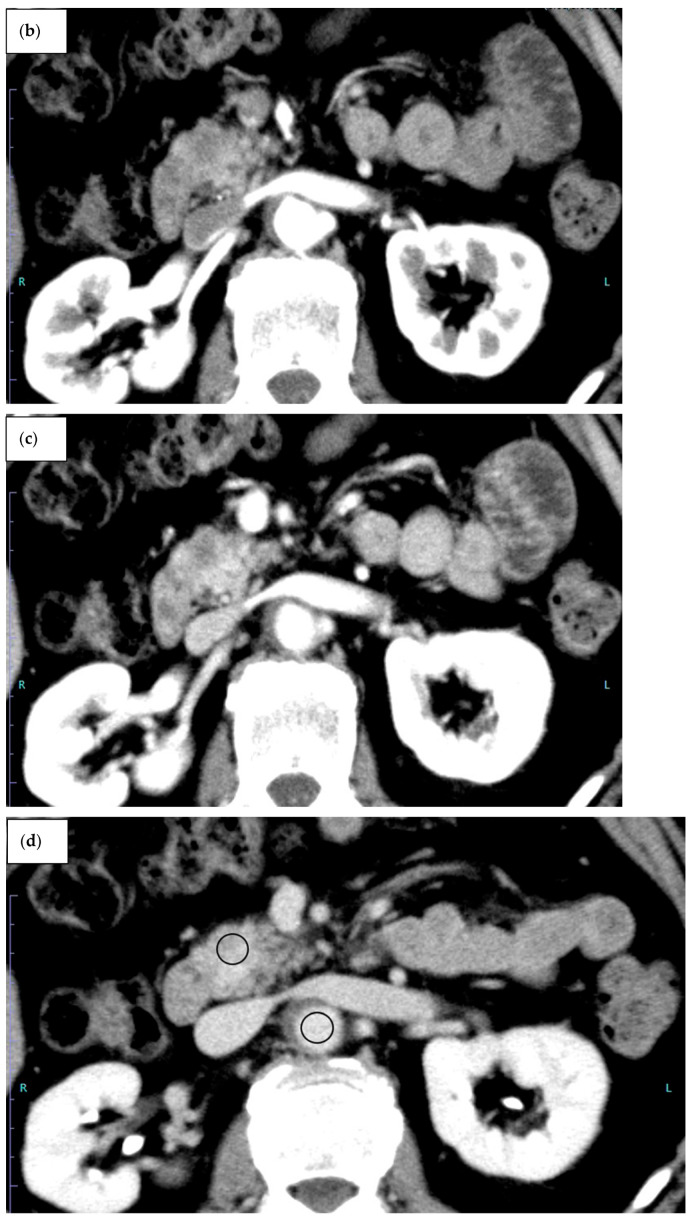

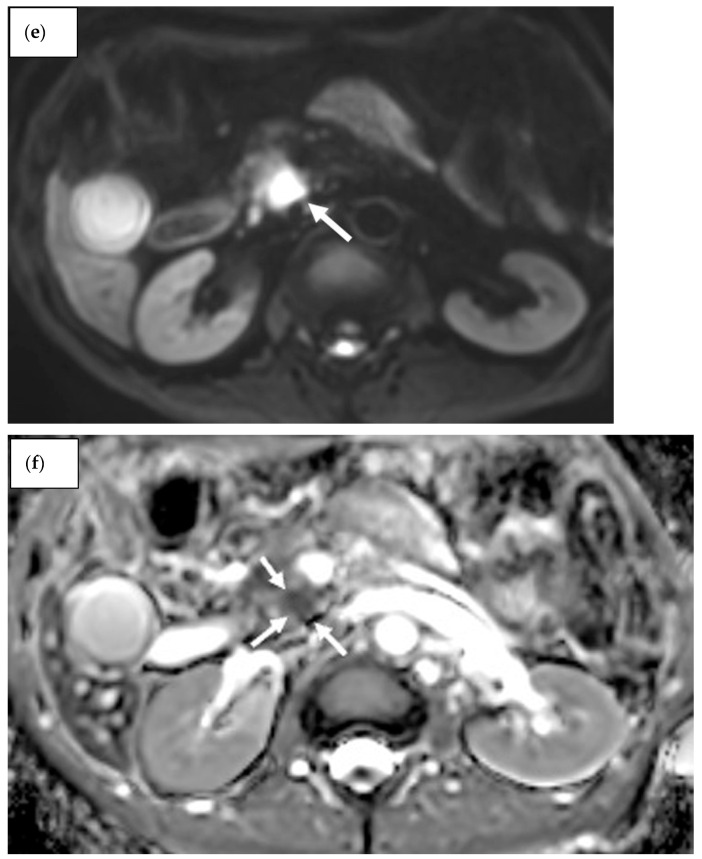
A 67-year-old man with pancreatic ductal adenocarcinoma (PDAC). (**a**–**d**) CT images pre-contrast (**a**) and during the pancreatic phase (**b**), portal venous phase (**c**), and equilibrium phase (**d**). Regions of interest (in black) are manually drawn on the PDAC region and abdominal aorta on pre-contrast (**a**) and equilibrium-phase (**d**) images. The calculated contrast enhancement was 38.5 HU and the extracellular volume fraction was 36.1%. (**e**) Diffusion-weighted imaging (b = 800 s/mm^2^) shows the PDAC region with high signal intensity (arrow). (**f**) The apparent diffusion coefficient is 0.79 × 10 ^−3^ mm^2^/s (arrows).

CT attenuation values (in Hounsfield units [HU]) of the AIP region in the AIP group, tumour region in the PDAC group, parenchyma in the head of the pancreas in the healthy group, and aorta in all groups were measured on pre-contrast and equilibrium-phase scans. CT attenuation values were measured using regions of interest (ROIs) of as large a size as possible, avoiding major vessels and the main pancreatic duct (Figure 2 and Figure 3).

The AIP region had a mean ROI size of 96.7 mm^2^, the PDAC region had a mean ROI size of 86.7 mm^2^, and normal parenchyma in the head of the pancreas had a mean ROI size of 54.0 mm. The ROI was significantly greater in the PDAC and AIP groups than in the healthy group (*p* < 0.001), but no significant difference was evident between the PDAC and AIP groups (*p* = 0.130). CT measurement was performed independently by two radiologists (H.S. and A.H.) with 8 years and 16 years of experience in abdominal CT, respectively. Values measured by the two observers were averaged to create the value used to represent each ROI. Both radiologists were blinded to clinical findings and histopathological results. The type of AIP was determined as the consensus decision of two radiologists (A.K. and K.M.), each with 6 years and 15 years of experience, respectively. These radiologists did not participate in the reading sessions. They classified the lesions as diffuse type and focal or segmental type. Focal or segmental AIP was defined as the involvement of one or two pancreas segments [9].

CE was calculated as follows: CE = CT attenuation (in HU) on equilibrium-phase CECT–CT attenuation on pre-contrast CT. ECV fraction was calculated as follows: ECV (%) = ΔHU_pancreatic region_/ΔHU_aorta_ × (100 − haematocrit [%]), where ΔHU_pancreatic region_ and ΔHU_aorta_ represent the CT attenuations on equilibrium-phase CECT minus the CT attenuation before contrast agent administration for the pancreatic lesion (AIP or PDAC regions) or pancreatic parenchyma and aorta, respectively.

### 2.4. Statistical Analysis

The intraclass correlation coefficient (ICC) was calculated to evaluate inter-observer agreement for CE and ECV fraction (ICC = 0.00–0.20, poor correlation; 0.21–0.40, fair correlation; 0.41–0.60, moderate correlation; 0.61–0.80, good correlation; and 0.81–1.00, excellent correlation) [28]. The distribution of all continuous variables was assessed for normality using the Kolmogorov–Smirnov test. CE and ECV fractions were analysed among the three groups using the Kruskal–Wallis test, followed by the Mann–Whitney U test with Bonferroni correction for continuous variables. Adjusted *p*-values were obtained manually by multiplying each raw *p*-value by the number of comparisons. For CE and ECV fractions, ROC curve analyses were carried out to assess the diagnostic performance in differentiating between AIP and PDAC using AUC calculations. Sensitivity, specificity, and accuracy in differentiating between the two groups were calculated using a threshold criterion determined by the highest Youden index. The DeLong test was used to compare the AUCs of CE and ECV fraction. In the AIP group, CE and ECV fractions were also compared for diffuse and focal or segmental types using the Mann–Whitney U test. Data were analysed using SPSS for Windows version 24.0 software (SPSS, Chicago, IL, USA) and MedCalc for Windows version 11.1.1.0 statistical software (MedCalc, Mariakerke, Belgium). Values of *p* < 0.05 were taken to indicate statistical significance.

## 3. Results

Inter-observer agreement was excellent, with ICCs of 0.87 (95% confidence interval [CI] 0.82–0.91) for CE and 0.86 (95%CI 0.79–0.90) for ECV fraction.

**Figure 4:**
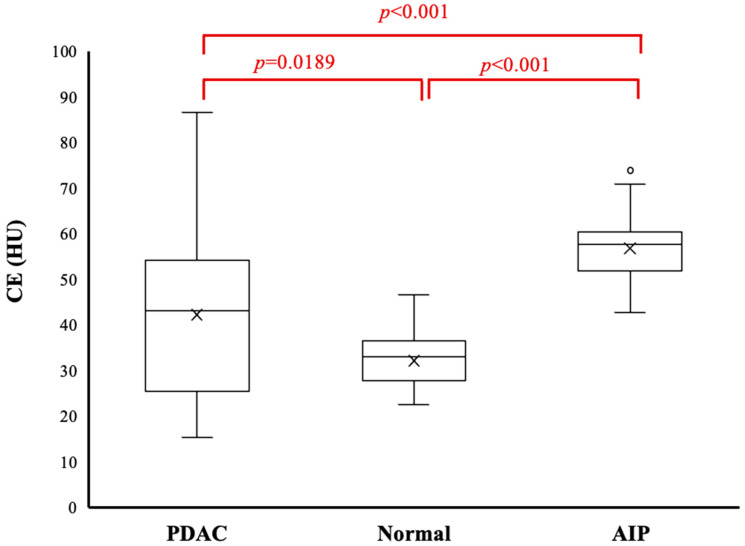
Boxplots for contrast enhancement (CE) of the pancreas in the three groups. Middle bar denotes the median. Cross marks within the box indicate group means. CE of the pancreas is significantly higher in the autoimmune pancreatitis group than in the healthy and pancreatic ductal adenocarcinoma (PDAC) groups (*p* < 0.001 each). A significant difference is also seen between the PDAC and healthy groups (*p* = 0.019).

CE and ECV fractions differed significantly between groups (*p* < 0.001 each). CE was significantly higher in the AIP group (56.8 ± 7.9 HU) than in the PDAC group (42.3 ± 17.0 HU, *p* < 0.001) or healthy group (32.2 ± 6.1 HU, *p* < 0.001). CE differed significantly between the PDAC group and healthy group (*p* = 0.0189) (Figure 4).

**Figure 5:**
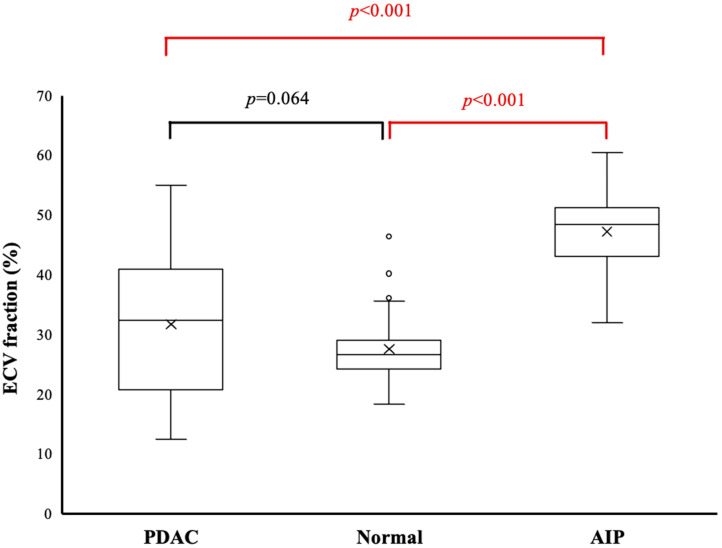
Boxplots for the extracellular volume (ECV) fraction of the pancreas in the three groups. Middle bar denotes the median. Cross marks within the box indicate group means. ECV fraction is significantly higher in the autoimmune pancreatitis group than in the healthy and pancreatic ductal adenocarcinoma (PDAC) groups (*p* < 0.001 each). No significant difference is evident between the PDAC and healthy groups (*p* = 0.064).

ECV fraction was significantly higher in the AIP group (47.2 ± 7.3%) than in the PDAC group (31.7 ± 12.0%, *p* < 0.001) or healthy group (27.5 ± 5.4%, *p* < 0.001). However, ECV fraction did not differ significantly between the PDAC group (31.7 ± 12.0%) and healthy group (49.2 ± 16.7%, *p* = 0.064) (Figure 5).

**Figure 6:**
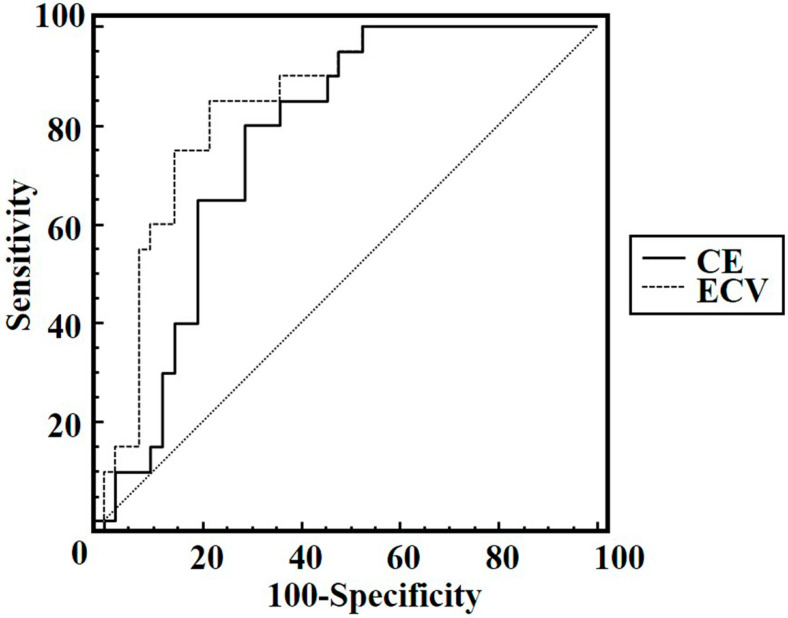
Results of receiver operating characteristic curve (ROC) analyses. ROC curve analysis for contrast enhancement reveals an area under the ROC curve of 0.78 (95%CI 0.66–0.88) and an optimal cutoff of ≥50.4 HU for diagnosing focal autoimmune pancreatitis (AIP) (80.0% sensitivity, 71.4% specificity, 74.2% accuracy). ROC curve analysis for extracellular volume fraction reveals an AUC of 0.86 (95%CI 0.74–0.93) and an optimal cutoff of ≥41.2% for diagnosing AIP (85.0% sensitivity, 78.6% specificity, 80.6% accuracy). The difference in AUCs between CE and ECV was not statistically significant (*p* = 0.083).

Using CE, differentiation between AIP and PDAC showed an AUC of 0.78 (95%CI 0.66–0.88), offering 80.0% sensitivity, 71.4% specificity, and 74.2% accuracy at an optimal cutoff of 50.4 HU. Using ECV fraction, AIP was able to be discriminated from PDAC with an AUC of 0.86 (95%CI 0.74–0.93), with 85.0% sensitivity, 78.6% specificity, and 80.6% accuracy at an optimal cutoff of 41.2%. However, the difference in AUCs between CE and ECV was not statistically significant (*p* = 0.083) (Figure 6).

In the AIP group, no significant differences in CE (56.7 ± 8.2 HU vs. 56.9 ± 8.1 HU, *p* = 1.000) or ECV fraction (48.0 ± 5.6% vs. 46.6 ± 8.4%, *p* = 0.970) were found between diffuse and focal or segmental types.

## 4. Discussion

Recent advances in endoscopic ultrasound-guided fine needle aspiration/biopsy technology have substantially improved the diagnostic accuracy for AIP. However, its use in clinical practice remains limited [29]. Therefore, the development of advanced imaging modalities is essential to further enhance diagnostic performance.

The present study was conducted to determine whether ECV from CECT can help differentiate AIP from PDAC. We found that CECT estimates of ECV fraction differed significantly between AIP and PDAC groups. Applying the ECV fraction as determined by CECT could thus be clinically useful in differentiating AIP from PDAC. This suggestion is underlined by the excellent inter-observer agreement.

ECV fraction, as determined from equilibrium CECT or MRI, has been used to assess cardiac, hepatic, and pancreatic fibrosis [18,19,20,21,22,23]. However, to the best of our knowledge, no reports have described differentiating AIP from PDAC using the ECV fraction from CECT.

In our study, CE was significantly higher in both the AIP and PDAC groups than in the healthy group. AIP is recognised as being associated with periductal lymphoplasmacytic infiltration, obliterative phlebitis, and storiform fibrosis. PDAC is recognised as being characterised by dense fibrosis caused by activated fibroblasts and collagen deposition within the tumour [30]. Advanced fibrosis in the tissue enhances the contrast effect during the delayed phase of CECT [13]. Therefore, the AIP and PDAC groups may have had higher CE values than the healthy group. Our study showed significantly higher CE in the AIP group than in the PDAC group. Muhi et al. found that mean CT attenuation values were significantly higher in AIP than in PDAC in the delayed phases [5]. Kamisawa et al. reported that all AIP patients had lesions delineated in the delayed phase, while PDAC was delineated in 61% of patients [31]. A perfusion CT study revealed that the peak enhancement intensity of mass-forming chronic pancreatitis was 16.5% higher compared to that of pancreatic adenocarcinoma [15]. As these results show, AIP often has higher CT attenuation values than PDAC in the delayed phase, which may be why the CE was significantly higher in AIP than in PDAC in our results. Nevertheless, CE depends on the CT scan protocol and patient characteristics, as well as the vascularity of the target tissue [13].

ECV fraction has been validated as a robust and stable quantitative parameter, independent of many technical and physiological confounders [32]. ECV fraction is reportedly associated with fibrosis of the heart and liver [18,19,20,21,22,23]. Recently, the ECV fraction was reported as a useful imaging biomarker for predicting treatment response and prognosis after chemotherapy in PDAC patients [24,25,26]. Further, the ECV fraction in PDAC has been reported to correlate strongly with volume transport constant (Ktrans) and the extravascular extracellular volume (Ve) obtained by DCE-MRI [26]. In our study, ECV fraction was significantly higher in the AIP group than in the healthy group. Advanced fibrosis enlarges the extravascular extracellular space [18,19,20,21,22,23]. The higher ECV fraction may therefore be attributable to the larger extravascular extracellular space reflecting abundant fibrosis within AIP. The AIP group showed a significantly higher ECV fraction than the PDAC group. Although the exact reasons for the higher ECV fraction in the AIP group are difficult to determine, the following explanation is plausible. A previous study found no significant difference in extravascular extracellular space calculated from DCE-MRI between AIP and PDAC [16]. On the other hand, the intravascular cavity calculated from perfusion CT was reported to be larger in AIP than in PDAC [15,17]. An increase in intravascular space might thus lead to a higher ECV fraction within AIP.

Differentiation between focal AIP and PDAC is reportedly difficult [2,4,5,6,11]. Direct comparison of focal AIP with PDAC was impossible due to the small number of patients with focal AIP in the present study. However, no significant differences between diffuse and focal or segmental types of CE or ECV were seen in the AIP group. These results therefore suggest that CE and ECV fractions may be clinically useful in differentiating focal AIP from PDAC.

Our study showed several limitations. First, this single-centre study included only a small number of patients with AIP. Further studies with larger sample sizes are necessary to confirm the results. Second, the ECV fraction was measured at our institution using a 210 s delay for equilibrium-phase images. Previous reports have shown that setting the equilibrium phase beyond 10 min improves the accuracy of ECV [18,21,33,34]. On the other hand, reports have described obtaining good accuracy for the ECV fraction even when the equilibrium phase was set at 180 or 210 s [23,35]. Measuring the ECV fraction using the routine equilibrium phase would certainly be more feasible in terms of clinical practice and technical success.

In conclusion, while the ECV fraction derived from CECT demonstrated a higher AUC than contrast enhancement, the difference did not reach statistical significance. Therefore, although a high ECV fraction may be indicative of AIP, its potential diagnostic value should be interpreted with caution, and further validation in larger cohorts is necessary.

## Figures and Tables

**Table 1:** Patient characteristics.

	PDAC (*n* = 42)	Healthy (*n* = 39)	AIP (*n* = 20)	^b^ *p* Value
^a^ Age (y)	70.1 ± 8.2	63.1 ± 17.3	64.5 ± 9.3	0.3
Sex				0.577
Man	28 (66.7)	22 (56.4)	14 (70.0)	
Woman	14 (33.3)	17 (43.6)	6 (30.0)	
^a^ IgG4	38.4 ± 30.2		893.9 ± 1066.9	*p* < 0.001
^a^ CA19–9	5364.7 ± 11,679.7		25.7 ± 31.6	*p* < 0.001
^a^ Tumour size (mm)	31.5 ± 15.3			
Tumour location				
Head	18 (42.9)			
Body	6 (14.3)			
Tail	18 (42.9)			
Type of AIP				
Focal/segmental			8 (40)	
Diffuse			12 (60)	

PDAC, pancreatic ductal adenocarcinoma; AIP, autoimmune pancreatitis. Numbers in parentheses represent percentages. ^a^ Data are given as mean ± standard deviation. ^b^ *p* < 0.05 were taken as statistically significant.

**Table 2:** Imaging parameters for all CT scanners.

	Tube Voltage (kVp)	Maximum Allowable Tube Current (mA)	Detector Row Configuration (mm)	Gantry Rotation Speed (s)	Helical Pitch	Matrix	Thickness and Reconstruction Interval (mm)
Aquilion Prime SP	120	600	40 × 1.0	0.5	0.825	512 × 512	5 and 5
Aquilion Prime SP/i Edition	120	600	80 × 0.5	0.5	0.813	512 × 512	5 and 5
Aquilion 64	120	600	32 × 1.0	0.5	0.844	512 × 512	5 and 5
Light speed Ultra 16	120	440	16 × 1.25	0.5	1.375 or 1.75	512 × 512	5 and 5

## Data Availability

The datasets generated and/or assessed in the current study are not publicly available due to the private, sensitive nature of the data. However, they are available from the corresponding author upon reasonable request.

## Abbreviations

The following abbreviations are used in this manuscript:

AIP	autoimmune pancreatitis
AUC	area under the receiver operating characteristic curve
CE	contrast enhancement
CECT	contrast-enhanced computed tomography
DCE	dynamic contrast-enhanced
CI	confidence interval
ECV	extracellular volume
ICC	intraclass correlation coefficient
MRI	magnetic resonance imaging
PDAC	pancreatic ductal adenocarcinoma
ROI	region of interest
Ktrans	volume transport constant
Ve	extravascular extracellular volume
